# Supplementary material for: Measuring respectful maternal and newborn care in Nepal: Comparing linked observation and interview data- prospective cohort study
Source: PLOS Glob Public Health. 2025 Jul 17;5(7):e0003933. doi: 10.1371/journal.pgph.0003933 (PMC12270105; doi:10.1371/journal.pgph.0003933)
Supplement: S1 Table — (DOCX) [file pgph.0003933.s002.docx]

S1 Table. Background characteristics comparison between selected and unselected samples (Observation)

| **Indicators** | **Selected Population Observation and Interview (N=22832)** | **Unmatched Population with Observation**  **(N=5979)** |
| --- | --- | --- |
| **Age (Mean±SD)** | **23.79±4.08** | **24.3±4.3** |
| < 20 years | 1634 (7.2) | 396(6.6%) |
| 20- 35 years | 20994 (91.9) | 5524(92.4%) |
| >35 years | 204 (0.9) | 59(1.0%) |
| **Ethnicity** |  |  |
| Dalit | 3034 (13.3) | 660 (11.0%) |
| Janajati | 6749 (29.6) | 1975 (33.0%) |
| Madhesi | 3212 (14.1) | 1026 (17.2%) |
| Muslim | 752 (3.3) | 138 (2.3%) |
| Chhetri/Brahmin | 9085 (39.8) | 2180 (36.5%) |
| **Parity** |  |  |
| 0 previous births | 12118 (53.1) | 1720(54.9%) |
| 1 previous birth | 7800 (34.2) | 858 (27.4%) |
| >2 births | 2,914 (12.7) | 555(17.7%) |
| **Education** |  |  |
| No primary education | 2879(12.6) | NA |
| Primary education | 4329 (19.0) | NA |
| ≥Secondary education | 15,624 (68.5) | NA |
| **Wealth index** |  |  |
| Poorest | 7866(34.5) | NA |
| Poorer | 4624(20.3) | NA |
| Middle | 2016(8.8) | NA |
| Richer | 3457(15.1) | NA |
| Richest | 4869(21.3) | NA |
