# Supplementary material for: Measuring respectful maternal and newborn care in Nepal: Comparing linked observation and interview data- prospective cohort study
Source: PLOS Glob Public Health. 2025 Jul 17;5(7):e0003933. doi: 10.1371/journal.pgph.0003933 (PMC12270105; doi:10.1371/journal.pgph.0003933)
Supplement: S2 Table — (DOCX) [file pgph.0003933.s003.docx]

**S2 Table**. Background characteristics comparison between selected and unselected samples (Interview)

| **Indicators** | **Selected Population Observation and Interview (N=22832)** | **Unmatched Population with Interview**  **(N=10967)** | **p-value** |
| --- | --- | --- | --- |
| **Age (Mean±SD)** | **23.79±4.08** | **24.8±4.4** | <0.0001* |
| < 20 years | 1634 (7.2) | 555(5.1%) |  |
| 20- 35 years | 20994 (91.9) | 10204(93.0%) |  |
| >35 years | 204 (0.9) | 208(1.9%) |  |
| **Ethnicity** |  |  | 0.047 |
| Dalit | 3034 (13.3) | 1486 (13.5%) |  |
| Janajati | 6749 (29.6) | 3307(30.2%) |  |
| Madhesi | 3212 (14.1) | 1492(13.6%) |  |
| Muslim | 752 (3.3) | 301 (2.7%) |  |
| Chhetri/Brahmin | 9085 (39.8) | 4381 (39.9%) |  |
| **Parity** |  |  | <0.0001 |
| 0 previous births | 12118 (53.1) | 5176(47.2%) |  |
| 1 previous birth | 7800 (34.2) | 3979 (36.3%) |  |
| >2 births | 2,914 (12.7) | 1812(16.5%) |  |
| **Education** |  |  | <0.0001 |
| No primary education | 2879(12.6) | 1585(14.5%) |  |
| Primary education | 4329 (19.0) | 2033(18.5%) |  |
| ≥Secondary education | 15,624 (68.5) | 7349 (67.0%) |  |
| **Wealth index** |  |  | <0.0001 |
| Poorest | 7866(34.5) | 4031(40.7%) |  |
| Poorer | 4624(20.3) | 1962(19.8%) |  |
| Middle | 2016(8.8) | 571(5.8%) |  |
| Richer | 3457(15.1) | 1395(14.1%) |  |
| Richest | 4869(21.3) | 1940(19.6%) |  |

Independent Samples test*, Chi-Square Test
