# Supplementary material for: Measuring respectful maternal and newborn care in Nepal: Comparing linked observation and interview data- prospective cohort study
Source: PLOS Glob Public Health. 2025 Jul 17;5(7):e0003933. doi: 10.1371/journal.pgph.0003933 (PMC12270105; doi:10.1371/journal.pgph.0003933)
Supplement: S3 Table — (DOCX) [file pgph.0003933.s004.docx]

**S3 Table**. Domains, Data collection method and item, and percentage agreement between observation and interview by hospital

| Hospital A |  |  |  |  |  |
| --- | --- | --- | --- | --- | --- |
| Domains | Observation (9486) | Exit Interview (9486) | Observation | Interview | k, LOA |
| Consent / counselling | Consent taken | Informed about routine care | 84.8% | 96.5% | 82.7% |
| Respect / dignity | Treated with respect and dignity | Treated with respect and dignity | 98.0% | 98.6% | 96.6% |
|  | Companion during birth | Companion of choice during Labour and Birth | 2.3% | 10.9% | 87.3% |
| Care provision | Infant-women skin-to-skin contact | Infant-Women skin-to-skin contact | 3.2% | 8.8% | 88.6% |
|  | Initiation of breastfeeding | Initiation of breastfeeding | 7.9% | 6.9% | 86.5% |
|  | Infant kept warm | Infant kept warm | 93.3% | 94.6% | 91.5% |
|  | Umbilical care | Umbilical care | 87.0% | 15.9% | 36.6% |
| Hospital B |  |  |  |  |  |
| Domains | Observation (5416) | Exit Interview (5416) | Observation | Interview | LOA |
| Consent / counselling | Consent taken | Informed about routine care | 25.0% | 69.0% | 39.1% |
| Respect / dignity | Treated with respect and dignity | Treated with respect and dignity | 93.6% | 99.3% | 92.8% |
|  | Companion during birth | Companion of choice during Labour and Birth | 16.6% | 11.1% | 15.3% |
| Care provision | Infant-women skin-to-skin contact | Infant-Women skin-to-skin contact | 1.8% | 12.6% | 86.3% |
|  | Initiation of breastfeeding | Initiation of breastfeeding | 8.3% | 2.5% | 89.4% |
|  | Infant kept warm | Infant kept warm | 94.5% | 97.1% | 93.7% |
|  | Umbilical care | Umbilical care | 77.6% | 2.6% | 50.5% |
| Hospital C |  |  |  |  |  |
| Domains | Observation (7930) | Exit Interview (7930) | Observation | Interview | LOA |
| Consent / counselling | Consent taken | Informed about routine care | 17.7% | 64.1% | 44.7% |
| Respect / dignity | Treated with respect and dignity | Treated with respect and dignity | 96.5% | 99.4% | 95.8% |
|  | Companion during birth | Companion of choice during Labour and Birth | 2.4% | 1.3% | 96.4% |
| Care provision | Infant-women skin-to-skin contact | Infant-Women skin-to-skin contact | 6.2% | 89.9% | 13.3% |
|  | Initiation of breastfeeding | Initiation of breastfeeding | 17.2% | 22.6% | 79.9% |
|  | Infant kept warm | Infant kept warm | 96.4% | 98.5% | 95.8% |
|  | Umbilical care | Umbilical care | 93.9% | 36.4% | 89.8% |
