# Supplementary material for: Measuring respectful maternal and newborn care in Nepal: Comparing linked observation and interview data- prospective cohort study
Source: PLOS Glob Public Health. 2025 Jul 17;5(7):e0003933. doi: 10.1371/journal.pgph.0003933 (PMC12270105; doi:10.1371/journal.pgph.0003933)
Supplement: Attachment — Submitted filename: Report to PGPH 7.docx [file pgph.0003933.s007.docx]

Dear Editor,

Thanks for the opportunity to review this manuscript. My recommendations are below.

1) The title should meet the SMART requirements.
2) In the Abstract part it should include the sample size, sampling techniques, study period and area, it should replace discussion part by conclusion
3) In conclusion part, You have to summarize your conclusion based on findings
4) What about your recommendations based on findings
5) References should be up to date, old references example reference number 32.

Best regards
