# Supplementary material for: Measuring respectful maternal and newborn care in Nepal: Comparing linked observation and interview data- prospective cohort study
Source: PLOS Glob Public Health. 2025 Jul 17;5(7):e0003933. doi: 10.1371/journal.pgph.0003933 (PMC12270105; doi:10.1371/journal.pgph.0003933)
Supplement: Attachment — Submitted filename: Response Letter 24.10.24.docx [file pgph.0003933.s008.docx]

**24.10.2024**

**Response Letter**

Thank you for your thorough review and valuable comments on our manuscript. We appreciate the time and effort you have taken to provide us with your insightful feedback. Below, we address each of your comments and concerns:

Response to editor’s comments

Comment 1- 'Respectful care' domains: the domains presented are not well situated within the WHO standards and other major guidelines and frameworks for quality of care, and it is not clear how these domains align with the articles provided as references. A more clear typology and more detail on the links between the domains measured here and the substantial literature on maternal care quality is required. In particular, care provision should be situated under Experience of Care (per WHO framework) or technical quality of care. It is particularly important to distinguish between experience of care measures and technical care delivery measures when approaching a measurement analysis. Whether the other two domains can both be described as respectful care vs the broader 'experience of care' grouping used in WHO standards should also be considered and appropriately situated within the literature.

Response- We agree that the WHO standard of care does mention the respectful care domain. In this analysis, the respectful care domain not only includes the experience of care but also the provision of care. Respectful is one dimension of quality of care and require measurement of both experience and provision of care. We have provide our rationale in page 3, lines 19-31.

Comment 2- Purpose of the analysis: This article rests on a body of literature comparing observed vs. self-reported technical care indicators (typically attempting to validate the reported indicator against the direct observation: <https://pubmed.ncbi.nlm.nih.gov/33765956/>) and a more recent literature on experience of care measurement. Assessments of experience measures have differed in whether to consider the patient perspective (<https://pubmed.ncbi.nlm.nih.gov/31384074/>, <https://pubmed.ncbi.nlm.nih.gov/37479486/>) or the direct observation (<https://www.joghr.org/article/38745-measurement-of-respectful-maternity-care-in-exit-interviews-following-facility-childbirth-a-criterion-validity-assessment-in-nigeria>). (Disclosure - i am a co-author on Mehrtash et al.). This makes it particularly essential to address the reason for this research study and how the analysis matches that. The authors primarily refer to agreement and concordance, suggesting neither measurement approach is favored as more accurate, although the introduction refers to the need to validate self report. Any of these approaches could be appropriate, but a clear choice, justification, and alignment of methods accordingly is required.

Response- This not a validity study, where one tool is compared against another gold standard measurement. In the EN-BIRTH study, <https://pubmed.ncbi.nlm.nih.gov/33765956/>, we did the validity assessment where we compared reporting in comparison to observation as gold standard. However, this study was not designed to compare reporting tool with the gold standard measurement. We assess the concordance or discordance of the two tools for respectful newborn care domain. We have added <https://gh.bmj.com/content/5/Suppl_2/e004822.long> as reference where there was high level of disagreement between observation and reporting. We have also added the challenge of measuring experience of care <https://pubmed.ncbi.nlm.nih.gov/31384074/>, <https://pubmed.ncbi.nlm.nih.gov/37479486/>

Comment 3- Alignment of indicators: as noted in both reviews, a reliability analysis demands equivalent measurement between the two approaches; the items here do not all meet this standard. Please consider focusing the analysis only on indicators where a reasonable argument can be made for a common underlying action being measured.

Response- We have now aligned the indicator for consent and counseling in observation and reporting tool in table 2 and the results similarly.

Comment 4- Inclusion criteria: please include a comparison of characteristics of women whose data could be included in this analysis to those excluded (no match between observation and interview) at minimum as a supplement and consider any implications for your findings.

Response- We have now provided the comparison of the characteristic of women who were excluded in the analysis (S1Table1)

**Response to the first reviewer’s comments:**

**Comment 1.** The introduction should clearly articulate why the authors undertook the analysis. The stated aim is to evaluate the correlation between approaches, but why? What is to be learned or gained? My assumption would be the goal is to assess the reliability of patient report, but the many differences in measurement make this very difficult. If anything, this study demonstrates the challenges inherent in trying to capture and compare observed actions and respondent perceptions and could be useful in parsing out the contributing factors.

**Response**: We have provided the rationale on why there is a need to compare respectful maternal and newborn care domain using two different measurement approaches, i.e independent observation vs client interview. Respectful maternal and newborn care is relatively a new dimension on quality of care and there is no established methods respectful care. Studies to measure respectful maternal and newborn care have either used clinical observation or client exit interview. Both these tools have their biases and thus a correlation is to be established on how different domains of respectful maternal and newborn care is measured. This study aims to assesses the comparison of measurement for the particular respectful domain. We have further explained it in background section page 3, lines 19-31 and page 4, lines 1-2.

**Comment 2.** If the goal is to assess whether patients can perceive good or poor experience when it is observed to have occurred or not occurred, I have concerns about the extent to which the observation indicators and the exit interview questions measure the same constructs. The observation versus exit interview questions in the consent and counseling domain seem particularly different. For example, I am unclear how "was consent taken" as an observation question measures the same construct as "were you adequately informed about exams.". A woman may have been consented, but also may not have felt it was adequate information or that it was done for all interventions. If items do not measure the same construct, we would expect some degree of discordance simply from the instrument.

**Response:** We agree that the observation checklist and client exit interview were not designed to match each other for measuring quality of care. We had an independent observer and interviewer for collecting the information for the measures. The primary objective to the study was to independently assess quality of care measure using two different tools. Following the results of the primary study, in the post-hoc review, we mapped and matched the observation checklist and client exit interview for particular respectful maternal and newborn care domain.

We have removed the parameters regarding infant examination in women’s presence and concern discussed from the Table 4 consent to care section. This has also been revised at the result section page 11, line numbers 1 to 3.

**Comment 3.** In the respect/dignity domain, how do observers assess whether the woman was "treated with kindness and respect during delivery"? That assessment could fall prey to the same murky measurement issues as exit interviews. Similarly, the observers assessed if anyone accompanied the mother, while the exit interviewer asked about companion of your choice. Again, quite a difference (and peculiar that more women said they had a companion than was observed.).

**Response**: We agree with the pertinent comment on whether “treated with kindness and respect during delivery” might have both subjective bias during the interview. This measure needs a qualitative in-depth exploration of the kindness and respect during delivery. We have mentioned this as one of the research gaps in page 14, lines 28-31. Also, we agree the companion of choice during labour measured in the interview and companion during childbirth measured through observation is not the same, as clinical observation cannot assess the companion of choice rather who accompanied the women. So, we have mentioned it in page 16, lines 7-13.

**Comment 4.** In the care provision domain (which to me reads more like technical quality than user experience), the observation indicator does not mention immediate skin contact like the interview question, and immediate can mean different things to different respondents. Helpfully, the care provision domain questions have the most fidelity between observations and interviews in the questions asked but have the least concordance, so this does suggest the analysis may be picking up some real differences.

**Response:** Thank you for your insightful feedback regarding the need for more detail on how items were observed and how questions were asked, particularly for nuanced concepts such as respect. We appreciate your observation that this is critical for making meaningful comparisons, and we recognize the importance of further elaborating on potential factors contributing to discordance. In response to your suggestions, we had initially trained the independent observers and interviewers on the parameters and they reported the responses accordingly which would fall under our standards of the care provision.

**Comment 5.** The title phrase "associated practices and experiences" is not clear to me--associated with what? My suggestion would be something around concordance of observed versus patient-reported measures of user experience, but I leave this to the authors' and editor's discretion.

**Response**: Thank you for the kind suggestion. We have updated the title and have removed the sentence associated practices and experiences.

**Comment 6.** It's unclear which of these clinical items (in the care provision section in particular) is observable by women in the study. Umbilical care had the lowest agreement--for example, is it possible that the observation team was able to see this done while mothers were not?

**Response**: Yes. We agree that the high discordance on umbilical care might be that women are not aware or informed on when the umbilical cord is being clamped. This provide a major information that women are not aware on what care newborn are receiving after birth. We have mentioned this in page 15, lines 1 to 7.

**Comment 7:** How were primary study participants selected for observations versus interviewing versus both? Were all respondents interviewed? Fig 1 suggests no. I am wondering whether the sample of women with both an observation and an interview could be different from the sample of women with only an observation, and how. This is mentioned in the limitations but details are not given.

**Response:** Thank you for the question. All women who were observed during deliveries were not interviewed. The interview took place at the time of discharge so there were mothers who were observed but missed to be interviewed. We have also mentioned the number of women whose observation and interview did not match and were excluded from the analysis.

**Comment 8**: What was the context in which women were interviewed? Given how many interview-reported items are higher than observed items, I wonder about the extent of courtesy bias, a common issue with exit interviewing.

**Response**: Yes, we agree with the point of detection bias as the interview were taken at the postnatal ward where there is limited space for privacy and adding into that the study included high volume public hospitals which often has a crowded environment, which could bias the statement of the respondent.

**Comment 9**: The fig 2 plots are a bit hard to read. It looks like there may be an extra datapoint in fig 2a, and it's unclear in 2a what the "average 57.9%" text refers to. Fig 2b has different labels and fig 2c has none.

**Response:** The figure 2a, 2b and 2c are now updated. The indicator regarding concern discussed and infant examination in women’s presence has now been excluded from the consent and counselling plot 2a. The overall Bland Altman plot analysis has been updated, we have mentioned the mean differences between observation and interview parameters.

**Response to the second reviewer's comments.**

Comment 1. Add justifications for why "observations" and patient "experiences" need to correlate.

**Response**: There is a need to correlate “observations” and “interview” for respectful care, as measuring the respectful care empirically has many challenges, as experiences can be subjective and dependent on expectations. See page 3 lines 23-30. “Previous studies have found that some types of disrespect and abuse are observed, but not reported, while other types are reported but not observed. Research further shows that many newborns receive below the standard care, both in terms of provision of care and experiences of care. Since maternal and newborn experiences are interlinked and can affect each other, there is a need to measure the experience of maternal and newborn care through interviews with women and through observation, and ideally in a way that can compare the findings from each type of data collection.”

**Comment 2.** Describe the components of consent that were measured during "observation", if available. Did it include any of the components measured during "experiences"?

**Response**: The operational definition for measuring “consent of care” through observation was “clinical care provider took consent and explained the procedure for care”. We realized that the questions which matches with “consent of care” in interview was “Were you adequately informed by the care provider about examinations, actions, and decisions taken for your care throughout the hospital stay?”

**Comment 3.** Comparisons of "experiences" and "observations" for consent and counseling do not seem appropriate. While the "observation" relates to consent, the "interview" is largely about counseling.

**Response:** We have updated the consent and counselling domain at table 4 and figure 2a,b,c. We have now kept feasible comparison for information about routine care and high-risk care.

**Comment 4:** Describe how you calculated k. Did you use the "observed" proportion as the expected?

Response: Thank you for the information, we have explained the k value at the “Mapping of observation checklist and interview questionnaires” section. Yes, we have used the observed proportions as actual agreement between the raters.

**Comment 5**: Figure 1 could be simplified by starting with 34,581 "interviewed" participants, followed by a subset of 30,601 that were "observed".

Response: Thank you for the comment. The number 30601 as a whole does not include the interviews as they were done in a separate time by independent collectors, The number 22832 is the combined population consisting both interview and observation so we mentioned clear process of selecting the final population for this study

**Comment 6:** Table 4. Following from the methodology above, it is not clear why you are using 47.3% for each of the "interview" components.

**Response**: Thank you for the information. Table 4 is now updated with suitable parameters for observation and interview.

**Comment 7:** Figure 2 (Bland Altman Plot). Better description of the figure is needed. There are 4 blue dots in 2a but only 3 measures are shown in Table 4.

**Response:** The bland Altman plot and Table 4 consent and the counselling domain is now revised with updated numbers. We have updated the description of the results as well in the result section line 13-19.

**Comment 8:** Describe the implications of using secondary data for analysis that are outside of its intended purpose.

**Response**: The implication of secondary data to correlate observations vs interview was that the observation and interview questions were not identical. We did not assess the reliability of the observation check with interview questions. There is further needed to align the indicators of interest with tools (observation checklist vs interview questions). We have explained it in page 16, in lines 8-13.

**Comment 9**: Could there be other reasons for poor concordance between "observed" and "interviewed" other than differences in wording?

**Response**: The other reason for poor concordance between observation checklist to interview questionnaire might be due to interviewer bias. The observations were conducted by trained research nurses, who might have detection bias in terms of observation of respectful care.
