# Supplementary material for: Measuring respectful maternal and newborn care in Nepal: Comparing linked observation and interview data- prospective cohort study
Source: PLOS Glob Public Health. 2025 Jul 17;5(7):e0003933. doi: 10.1371/journal.pgph.0003933 (PMC12270105; doi:10.1371/journal.pgph.0003933)
Supplement: Attachment — Submitted filename: Updated Response Letter 16.3.2025.docx [file pgph.0003933.s009.docx]

16.03.2025

**Response Letter**

**Dear Dr. Collins Otieno Asweto, PhD
Academic Editor
PLOS Global Public Health**

Thank you for your thorough review and valuable comments on our manuscript. We appreciate the time you have taken to provide us with your insightful feedback. Below, we address each of your comments:

**Response to the first reviewer’s comments:**

Reviewer #1: 

**Comment 1:** Centrally, the background section still does not articulate why comparing these methods is a worthwhile exercise, though I think that it is. What do we learn from his comparison and why conduct it? The edit from "aim" to "opportunity" dilutes the purpose further in my opinion. This should be a simple fix but I think it's critical. The response to comment 2 from the editor is insufficient.

**Response:** We have provided the justification for this study in background and made relevant changes in page 3, lines 7-9 as “This shift underscores the importance of ensuring not only the care provided but also that it is delivered in a way that respects and supports women and their newborns.” in page 3 lines,12-16 as “ many women experience mistreatment during labor, including inadequate information, lack of consent, obstructing the presence of a birth companion, and withholding the provision of care without the consent of the woman [8, 9]. These negative experiences can discourage women from seeking health care facility for childbirth, ultimately affecting maternal and newborn health outcomes”. In page 3, lines 21-22 as “due to resource constraints including shortage of qualified health professionals, limited equipment, outdated facility and gaps in essential services”. In page 3, lines, 26-30 as “Previous studies have shown discrepancies between reported and observed experiences of care. This includes some types of disrespect and abuse are observed, but not reported, while other types are reported but not observed [16-18]. Despite increasing global recognition on the need to measure respectful care, research has primarily focused on maternal experiences with less attention given to newborns”.

In page 4, lines 7-8 as “there is limited evidence on how these methods compare in capturing maternal and newborn experiences simultaneously. In page 5, lines 15-16 as “As a part of this initiative, a prospective data collection system was designed to collect data on care” and page 4, lines 17-21 as “Using data from NePeriQIP, this study critically compares information collected through direct observation of women during labor, delivery and the immediate postpartum period with self-reported experiences from interviews. This comparison is necessary to determine whether different methods yield similar or different insights as well as to assess their implications for measuring and improving respectful maternal and newborn care. “.

**Comment 2:** I have the same comment about the section on "global public health implications." The section speaks to the importance of measuring experience for maternal and newborn health generally, but says little about the value of comparing different measurement approaches until the last sentence. This should be the central thrust of this section.

**Response:** We have added about comparing different measurement approaches in this part in page 16, lines 21-24 as “Existing assessments primarily focuses on provision of care, while experience related aspects such as consent and counseling, respect and dignity, and receipt of adequate information has largely been through qualitative studies rather than standardized epidemiological measurement”. In page 17, lines 3-5 as “With further research and pilot testing, the integration of multiple measurement approaches such as hospital exit interviews alongside periodic independent observations can enhance routine monitoring of experience of care” , and in page 17, lines 8-10 as “ our study underscores the need to measure these large-scale survey findings through cross-sectional observational studies to ensure accuracy and completeness in routine data collection”.

**Comment 3:** Certain domains that have been cut (eg, "concerns discussed" have been removed from table 2 and 4 but not deleted from the text in the discussion section (eg, paragraph 2). Please update.

**Response:** Thank you for the kind suggestion we have removed the sentence for concern discussed from the discussion section line 8-9.

**Comment 4:** The abstract specifically mentions how experience of care is difficult to validate. However, in their response, the authors make explicit this is not a validation study. I suggest removing this language if the study does not intend to validate a measurement approach.

**Response:** Thank you for the kind suggestion we have removed in page 2, line 4 of abstract section.

**Response to the third reviewers comment**

Reviewer #3:

**Comment 1**:  The title does not fully provide a clear picture of the study. What is the importance of comparing the observation and interview data? The title is hanging and is not complete. Is it to assess or measure the quality of care with regards to "respectful maternal and newborn care". It can be rephrased.

**Response:** We have added measuring “Measuring respectful maternal and newborn care in Nepal…”

**Comment 2** :Was the Nepal perinatal quality improvement program (NePeriQIP) a study or a national program? It is referenced as a program on page 4 lines 6-7. It is also referenced as a study on the same page under Methodology, line 16, and a project under line 18. There is need to clearly separate the NePeriQIP from the secondary analysis (basis of this manuscript) in the methodology.

**Response:** Thank you for the concern. NePeriQIP (Nepal Perinatal Quality Improvement Project) was a study implemented at public hospitals in Nepal across several province in coordination with the government stakeholders, the data collected from the NePeriQIP study was used to analyse the information. We have corrected the word program by project since it is the registered name of the study in line 6 of the background section. Similarly, we have slightly updated the sentence in page 4, lines 25-27 as “This is a secondary analysis of the perinatal dataset collected during NePeriQIP study in three public referral hospitals in Nepal for 18 months between April 2017 and October 2018”.

**Comment 3:** The results section narrative lines 9-14 are not clearly written. It would be best to document the results for the observation checklist alone, then have another paragraph to explain the interview questionnaires (as shown in the figure 1). The figure explains the information better than the narrative.

**Response:** Thank you for the kind suggestion. We have updated the narratives for the figure 1 in page 8, lines 14-22 as below “During the study period, a total of 30,601 women were eligible for clinical observation of whom 1780 did not provide consent leaving 28,821 women who consented and were observed. Among these 5979 had incomplete information or could not be matched with interview data, resulting in 22,832 women with matched observation records. Simultaneously, 34,581 women who delivered at the hospital were eligible for interview with 782 declining to participate leading to 33,799 women who consented and were interviewed. Of these 10,967 had incomplete information or could not be matched with observation data, ultimately yielding 22,832 women with matched interview records. Thus, a total of 22,832 cases had matching information from both observation and interviews and were selected for final analysis”

**Comment 4**: On the last page of the comments (comment 9). The response of "might be due to interviewer bias" needs to be qualified further.

**New Response:** In addition to the previous response research nurses despite being trained could have been subconsciously influenced by expectations or prior knowledge when assessing respectful care.

**Response to the second reviewer's comment**

**Comment 1**. The title should meet the SMART requirements.

**Response:** We have now improved it, adding the design.

**Comment 2**. In the Abstract part it should include the sample size, sampling techniques, study period and area, it should replace discussion part by conclusion

**Response**: We have replaced in page 2, lines 23-26 from abstract page 2 as “A significant differences existed between observed and self-reported measures of maternal and newborn care. This study highlights the need for a measurement approach that incorporates independent observations alongside self-reported data. There is also a need to further explore concordance between different sources for progress monitoring.”.

**Comment 3**: In conclusion part, You have to summarize your conclusion based on findings

**Comment 4**: What about your recommendations based on findings

**Response 3 and 4**: We have added conclusion and recommendation in page 17, line 14-25 as” This study identified significant variations between observed and self-reported measures of maternal and newborn care specifically in the domains of consent and counseling, respect and dignity, and provision of care. While the domain of respect and dignity showed high concordance between observations and exit interviews, consent and counseling, exhibited poor agreement, indicates discrepancies in how these aspects of care are perceived and reported. Similarly, within the provision of care domain, variations were noted in key indicators such as skin-to-skin contact umbilical care highlights challenges in accurate measurement. There is need to integrate standardized metrics and independent observational assessments to strengthen the accuracy of experience-of-care measurements and improve the monitoring and delivery of maternal and newborn health services. Further, this study highlights the need for a measurement approach that incorporates independent observations alongside self-reported data to improve maternal and newborn care assessments.”

.

**Comment 5:** References should be up to date, old references example reference number 32.

**Response :** We have added the latest reference

“ Basnet, O., Bhattarai, P., Malla, H., Gurung, R., Paudel, P., Sunny, A.K., Målqvist, M. and Ashish, K.C., 2024. Assessment of economic burden due to COVID-19 pandemic during institutional childbirth in Nepal: before-and-after design. *BMJ Public Health*, *2*(2)”.
