# Supplementary material for: Measuring respectful maternal and newborn care in Nepal: Comparing linked observation and interview data- prospective cohort study
Source: PLOS Glob Public Health. 2025 Jul 17;5(7):e0003933. doi: 10.1371/journal.pgph.0003933 (PMC12270105; doi:10.1371/journal.pgph.0003933)
Supplement: Attachment — Submitted filename: Response letter.docx [file pgph.0003933.s010.docx]

3 June 2025

Response to Reviewer’s comments

Comment 1- In the abstract section you should describe those factors in relation to respectful maternity and newborn care

Response- We have now added the maternal and newborn care in the abstract as well as the study design in the abstract in lines 7 and 11.
